# Supplementary material for: Investigation of Electromagnetic Pulse Compaction on Conducting Graphene/PEKK Composite Powder
Source: Materials (Basel). 2021 Jan 30;14(3):636. doi: 10.3390/ma14030636 (PMC7866504; doi:10.3390/ma14030636)
Supplement: Supplementary file 1 [file materials-14-00636-s001.pdf]

materials

Article

Investigation of Electromagnetic Pulse Compaction on Conducting Graphene/PEKK Composite Powder

Quanbin Wang [1], Deli Jia [1], Xiaohan Pei [1], Xuelian Wu [2,*], Fan Xu [2,*], Huixiong Wang [2], Minghao Cao [2] and Haidong Chen [2]

[1] Department of Oil& Gas Production Equipment, Research Institute of Petroleum Exploration and Development, Xueyuan Road 20#, Beijing 100083, China; wqb_upc@petrochina.com.cn (Q.W.); jiadeli422@petrochina.com.cn (D.J.); peixh@petrochina.com.cn (X.P.)

[2] School of Mechanical Engineering, Jiangsu University, Xuefu Road 301#, Zhenjiang 212000, China; 2211903042@stmail.ujs.edu.cn (H.W.); 18356635336@163.com (M.C.); chd0527@163.com (H.C.)

* Correspondence: xlwu@ujs.edu.cn (W.X.); xufan-00@126.com (X.F.)

Electronic supporting information

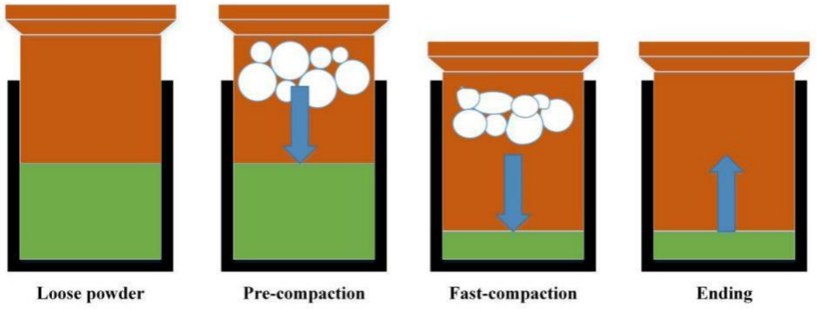

Figure S1. Working principle of electromagnetic assisted powder compaction.

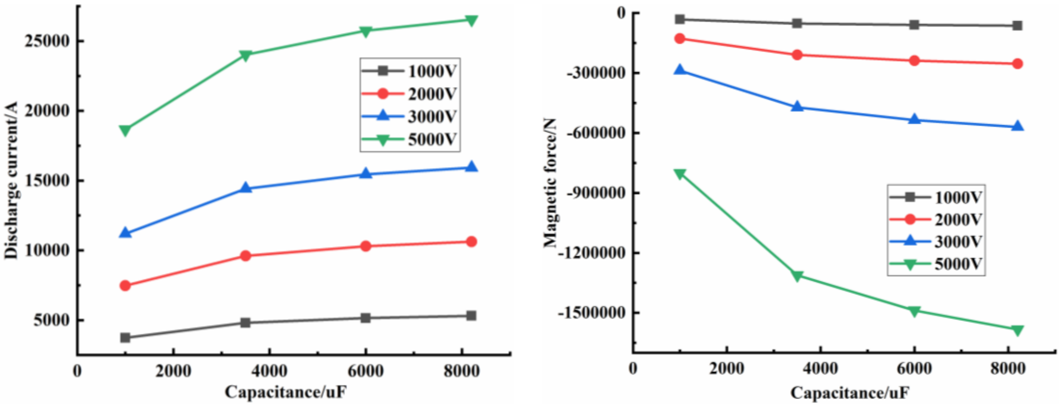

(**a**) (**b**)

Figure S2. Discharge current-Capacitance during the different discharge voltage. (**a**) Relationship between capacitance and current; (**b**) Relationship between capacitance and magnetic.

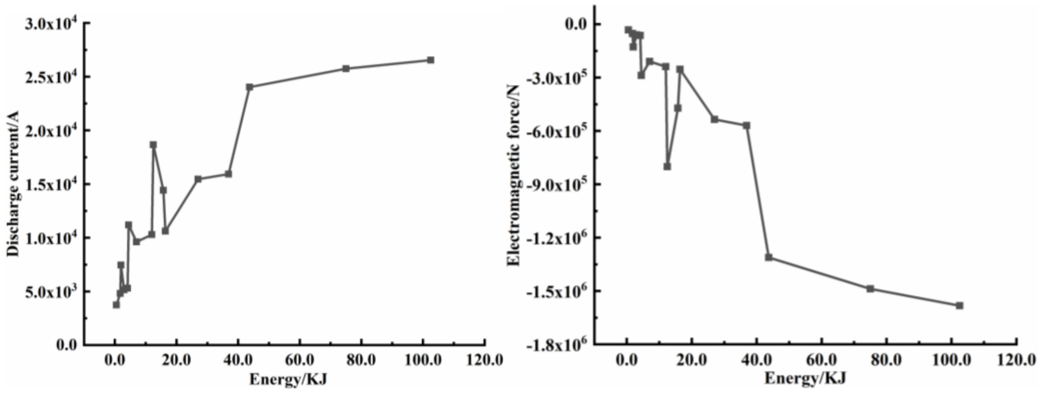

Figure S3. Relationship between energy and discharge current and voltage.

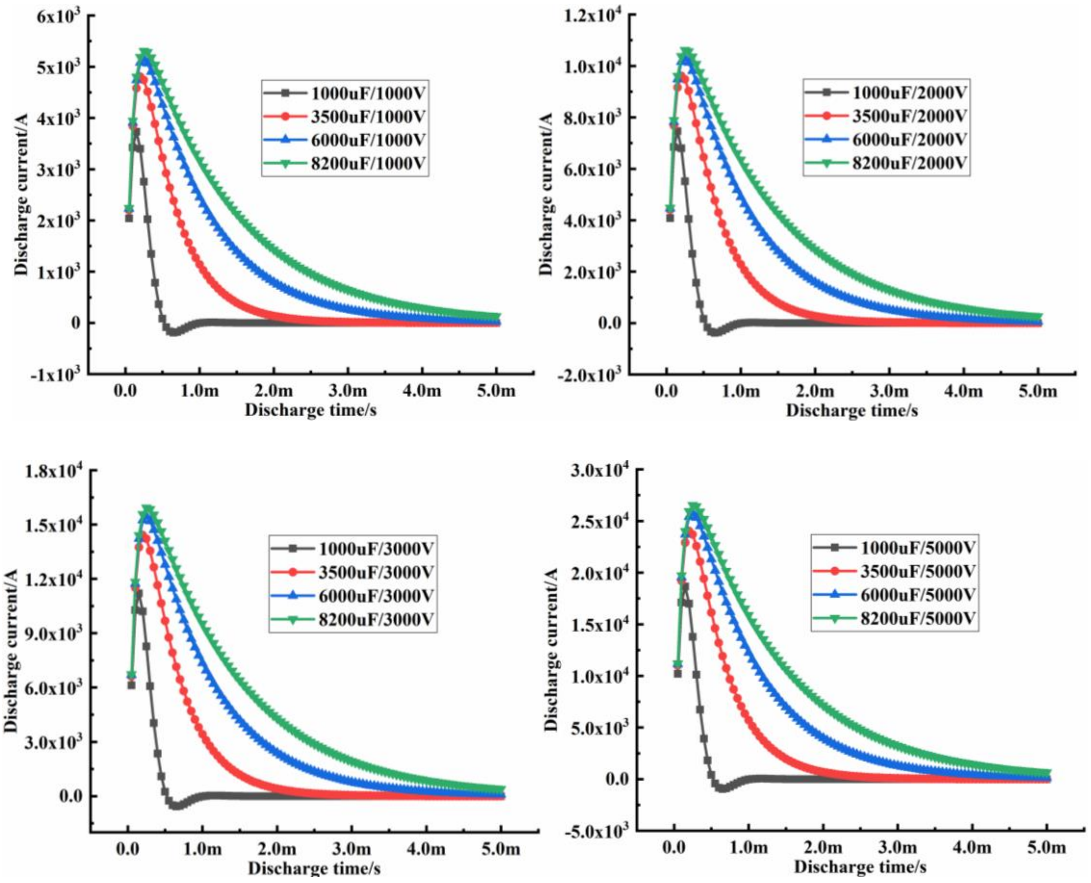

Figure S4. The different capacitance influence on discharge current.

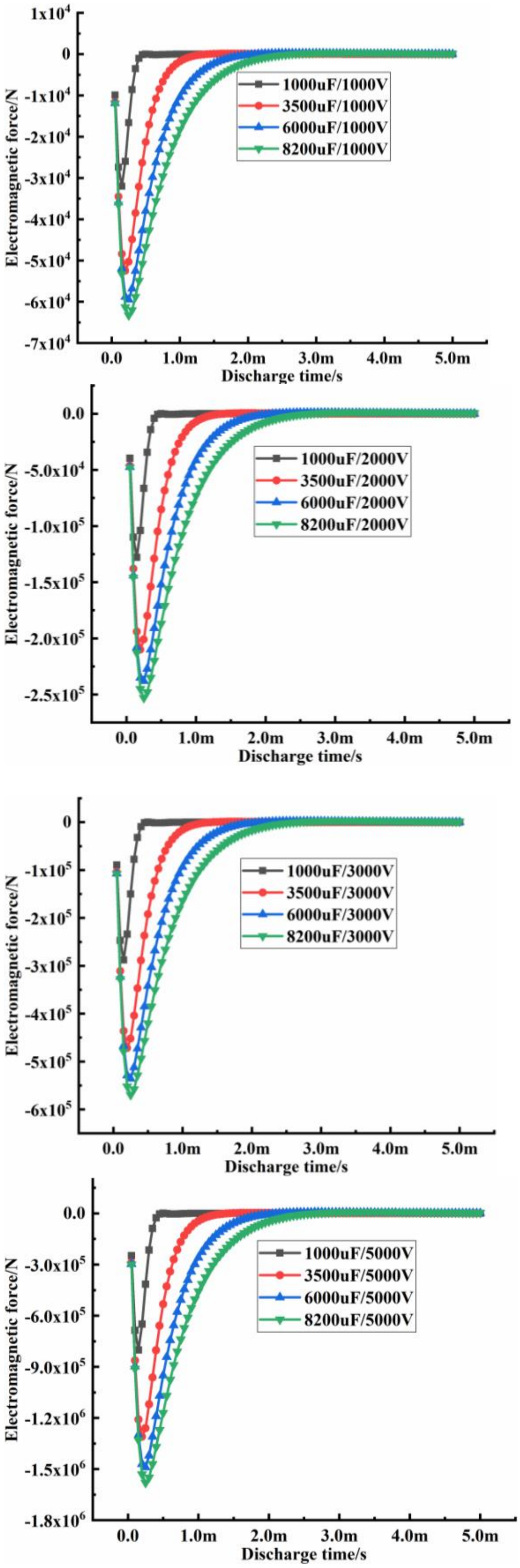

Figure S5. The different capacitance influence on electromagnetic force.

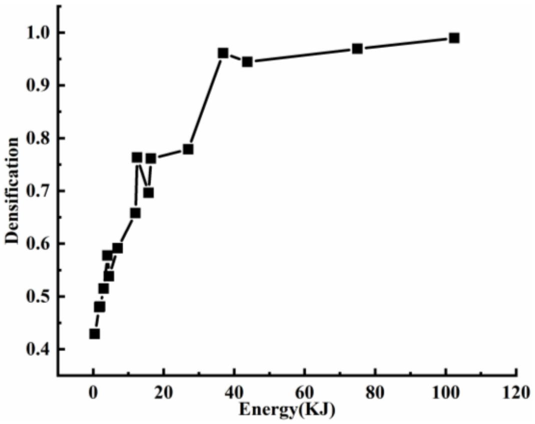

Figure S6. Influence of different energy on compaction densification.
